# Retraction: Modeling Dynamics of *Culex pipiens* Complex Populations and Assessing Abatement Strategies for West Nile Virus

**DOI:** 10.1371/journal.pone.0304217

**Published:** 2024-05-17

**Authors:** The PLOS ONE Editors

Following publication of this article [1], concerns were raised about the support for some of the parameter estimates and conditions used in the reported model, including the daily biting rate *β*, number of susceptible humans *H*_*S*_, susceptible female adult mosquito density *M*_*S*_, infectious female adult mosquito density *M*_*I*_, and susceptible bird density *B*_*S*_. In particular, a concern was raised that the daily biting rate range of 1–5 is too high, given that daily biting rate is typically calculated as the reciprocal of the duration of the gonotrophic cycle (in days), assuming one blood meal per gonotrophic cycle. The *PLOS ONE* Editors discussed the issues with the corresponding author KAP, who reviewed the model and carried out reanalyses revising some of the initial conditions and parameter values. Based on outcomes of the reanalyses, the corresponding author noted that the issues raised mainly affected the article’s findings related to West Nile virus (WNV) outbreak simulations.

The *PLOS ONE* Editors sought input about these issues from an external reviewer and two members of the Editorial Board.

The consulted experts generally agreed that the biting rate estimates used in [1] are higher than supported by previous literature (with the review article cited as reference [37] of the original article reporting values for *β* of 0.34–0.53), although it was also acknowledged that biting rate cannot be measured accurately, and there is a lack of data on the extent of multiple feeding (in which an individual mosquito requires multiple feeding episodes to obtain a complete blood meal).

A member of the Editorial Board reviewed the reanalyses and advised that there were unresolved concerns around insufficient reporting of the model fitting and the selection of some of the revised parameter values, including the revised biting rate. As such, the *PLOS ONE* Editors concluded that the reanalyses did not fully resolve the concerns, and that the results reported in the article related to modeling predictions in the presence of WNV outbreaks (including Fig 7, 8, and Table 7) are not supported.

In light of these concerns, the *PLOS ONE* Editors retract this article.

KAP did not respond to the final editorial decision. PN, CS, EJH, and GJH did not respond directly or could not be reached.
